# Occupational burnout among healthcare professionals in China: departmental differences, modifiable correlates, and public health intervention strategies

**DOI:** 10.1186/s12889-026-27711-8

**Published:** 2026-05-21

**Authors:** Cun-Bo Wu, Li-Chao Su, Dan-Lin Shen, Chuan-Zhi Zhang, Fu-Xin Lin, Zhang-Ya Lin

**Affiliations:** 1 Department of Neurosurgery, Neurosurgery Research Institute, The First Affiliated Hospital, Fujian Medical University, Fuzhou, China; 2 Department of Psychiatry, Chongqing Mental Health Center, Gele Mountain, Chongqing, China; 3 Mental Health Center, West China Hospital, Sichuan University, Chengdu, Sichuan Province China; 4 Department of Psychiatry, Jining Mental Health Center, Shandong, China; 5 National Center for Mental Disorders, West China Hospital, Sichuan University, Chengdu, Sichuan Province China

**Keywords:** Healthcare professionals, Occupational burnout, Departmental difference, Internal medicine, Psychiatry, Modifiable factors, Public health

## Abstract

**Background:**

Burnout is a critical threat to healthcare workforce stability and patient care quality globally, and it has evolved into a major public health concern in China due to unique systemic pressures. However, data on specialty-specific burnout differences—especially between high-stress fields like internal medicine and psychiatry—remain limited. This study aimed to characterize burnout prevalence, departmental variations, and factors associated with burnout among healthcare professionals in Jining, China, to inform targeted public health intervention hypotheses.

**Methods:**

A multicenter cross-sectional study was conducted among healthcare professionals from three tertiary hospitals in Jining. In total, 754 valid participants were included in the analysis. Burnout was measured using the Maslach Burnout Inventory (MBI), which showed good reliability in this study. One-way ANOVA and chi-square tests were used to compare burnout dimensions across departments and occupations. Univariate and multivariable logistic regression analyses were performed to identify factors associated with burnout.

**Results:**

The overall prevalence of burnout (total MBI score ≥ 50) was 7.3% (55/754). Internal medicine had the highest burnout scores across all dimensions, whereas psychiatry had the lowest, and differences remained significant after adjustment for hospital affiliation and occupation. Within psychiatry, physicians reported higher EE, whereas nurses showed higher DP and lower personal accomplishment.In multivariable analysis, independent factors associated with burnout included hospital affiliation, interpersonal relationship, introversion-extraversion, and neuroticism.

**Conclusions:**

Contrary to conventional assumptions, internal medicine was associated with higher burnout levels and psychiatry with lower levels after accounting for hospital and occupational confounders. Interpersonal relationship satisfaction, psychological traits (introversion-extraversion, neuroticism), and hospital affiliation were independently associated with burnout. Targeted interventions such as workload optimization in internal medicine and resilience-building practices from psychiatry may help mitigate burnout.

## Introduction

Burnout, defined as a syndrome of emotional exhaustion, depersonalization, and reduced personal accomplishment resulting from prolonged occupational stress, poses a substantial threat to the mental health of healthcare professionals and the quality of patient care globally [1]. Recognized by the World Health Organization in the International Classification of Diseases, 11th Revision (ICD-11) as a distinct occupational phenomenon [1], burnout has evolved from an individual concern to a critical public health challenge requiring systemic intervention. In clinical settings, burnout impairs professional judgment, diminishes empathy, and increases the risk of medical errors, with ripple effects affecting both providers and patients. For healthcare organizations, consequences include elevated staff turnover, reduced productivity, and increased healthcare costs, further straining already burdened systems.

Global research confirms healthcare as a high-risk field for burnout. In high-income countries, over 50% of physicians in the United States report at least one dimension of burnout, with rates exceeding 60% among frontline clinicians post-COVID-19 [2]. A systematic review of 36 international studies involving 17,364 midwives identified consistent risk factors: lack of professional autonomy, insufficient recognition, and chronic workload pressure [3], highlighting that intrinsic occupational factors often outweigh demographic variables in driving burnout. Similar patterns exist among nurses, particularly internationally educated nurses, for whom organizational barriers (poor practice environments, high patient-to-nurse ratios, cultural adaptation challenges) and individual factors (younger age, shorter tenure) synergistically increase burnout risk [4]. Beyond prevalence, international studies have elucidated multilevel determinants and consequences of burnout. European and North American research links electronic health record (EHR) burden to increased burnout, as administrative tasks divert time from direct patient care and erode professional fulfillment [5]. A cross-sectional study of 200 Romanian healthcare professionals found resident physicians most vulnerable (42% burnout rate), with ambulance staff facing heightened risk due to workplace-specific stressors; relationship status and work seniority emerged as key demographic correlates [6]. Intervention research has focused on organizational strategies (flexible scheduling, peer support programs, reduced administrative burden) and individual resilience-building, though evidence for sustained effectiveness remains mixed, underscoring the need for context-specific solutions [7].

In China, healthcare worker burnout has reached crisis proportions, driven by unique systemic pressures: high patient volumes, complex doctor-patient relationships, and uneven resource allocation. Epidemiological studies report prevalence rates of 66.5%–87.8% among mainland Chinese healthcare professionals, exceeding 90% in some primary care and public health settings [8]. A 2025 study of Chinese psychiatrists revealed distinct burnout profiles: significantly higher emotional exhaustion and cynicism, and lower personal accomplishment, compared with internal medicine physicians, with income dissatisfaction, interpersonal stress, and health status as key correlates [9]. This finding contrasts with some international observations [10], emphasizing the need for specialty-specific analyses within the Chinese context. Domestic research has also explored sociocultural and systemic influences on burnout. A multicenter cross-sectional study during the late 2022 Omicron outbreak found pandemic-related stressors (increased workload, infection risk, resource shortages) disproportionately amplified burnout among nurses and frontline staff [11]. While studies have identified demographic (age, work experience), occupational (department, job title), and psychological (self-efficacy, social support) correlates of burnout [12], critical gaps persist. Few studies have conducted adjusted head-to-head comparisons of burnout dimensions across multiple clinical specialties—particularly psychiatry versus general clinical settings—and the interplay between organizational support, cultural values, and burnout remains underexplored. Additionally, most domestic interventions focus on individual-level strategies, with limited attention to systemic reforms addressing root causes such as workload distribution and professional recognition [13].

This study addresses these gaps through a multicenter cross-sectional design involving three hospitals in Jining, Shandong Province. By assessing burnout across internal medicine(General internal medicine, cardiology, gastroenterology, respiratory medicine, nephrology, endocrinology, hematology), surgery(General surgery, orthopedics, neurosurgery, thoracic surgery, urology), obstetrics and gynecology, secondary specialties (Otolaryngology, Burn and Plastic Surgery, Dermatology, Traditional Chinese Medicine, General Practice), psychiatry, and other Specialties (Oncology, Pediatrics, Emergency, Intensive Care Unit(ICU), Infectious Diseases, Rehabilitation Medicine), and comparing physicians and nurses within these departments, we aim to identify specialty-specific and profession-specific burnout patterns. Further, by analyzing univariate and multivariable associations between burnout and demographic characteristics, psychological traits (neuroticism, extraversion), social support, and self-efficacy, we seek to elucidate key modifiable factors informing intervention strategies. Notably, this study provides rigorously adjusted comparisons that account for hospital and occupational confounding, which has been rarely undertaken in previous Chinese studies. Findings will enrich the global evidence base on healthcare worker burnout [14] and provide actionable insights for Chinese healthcare organizations to improve staff well-being, reduce turnover, and enhance care quality and safety—ultimately contributing to a more resilient workforce capable of meeting evolving healthcare demands [15].

## Methods

### Study design and participants

This multicenter cross-sectional study was conducted among healthcare professionals at three tertiary hospitals in Jining: Affiliated Hospital of Jining Medical University, Jining No.1 People’s Hospital, and Jining Mental Health Center. The study was performed in accordance with the Declaration of Helsinki. This was an anonymous, non-interventional, and minimal-risk questionnaire survey that did not collect any personally identifiable information.

Inclusion criteria were active clinical healthcare professionals (physicians and nurses) working in frontline departments who voluntarily agreed to participate. Exclusion criteria included auxiliary staff, administrative personnel, retired professionals, advanced practice providers in training (fellows, residents, nurses in training), individuals with severe physical illnesses, and those who declined participation or provided incomplete questionnaire data.

### Consent to participate

A clear explanation of the study purpose, voluntary participation, confidentiality, and the right to withdraw was provided on the front page of the questionnaire. All participants were informed that they could discontinue participation at any time without penalty. Completion and return of the questionnaire constituted implied informed consent.

### Sample size estimation

Sample size was calculated using the formula n = Z²P(1-P)/δ², where Z = 1.96 (95% confidence interval), *P* = 0.10 (estimated burnout prevalence from prior domestic studies [11]), and δ = 0.02 (margin of error). This yielded a minimum sample size of 865 participants. To account for non-response and incomplete data, the target sample size was expanded to approximately 2500. Data collection occurred from March 2018 to May 2018 via paper-based questionnaires distributed and collected on-site. Investigators received uniform training to ensure consistent administration, and participants completed surveys anonymously to minimize response bias. Of 2573 distributed questionnaires, 754 valid responses were included in the final analysis after excluding incomplete, duplicate, or ineligible forms. The final analytic sample (*n* = 754) was slightly lower than the calculated minimum (*n* = 865). However, post-hoc power analysis showed sufficient power (> 0.80) for the multivariable regression model, which was noted as a limitation.

### Data collection tools

Data were collected using a self-designed general information questionnaire and three validated scales. The general information questionnaire captured demographic characteristics (age, gender, marital status, educational level), occupational information (department, hospital, years of work, professional title), and life satisfaction (interpersonal relationships, income, family situation, physical health).

Occupational burnout was assessed using the Maslach Burnout Inventory (MBI)-Human Services Survey, a validated instrument comprising 22 items across three dimensions rated on a 0–6 Likert scale (0 = never, 6 = every day) [14]. Consistent with previous large-scale Chinese studies of healthcare burnout, occupational burnout was operationally defined as a total MBI score ≥ 50 [9, 10]. Emotional exhaustion (EE, 5 items) was defined as high with a cutoff score ≥ 27, indicating severe emotional depletion. Depersonalization (DP, 4 items) was defined as high with a cutoff score ≥ 10, reflecting significant cynicism toward patients. Reduced personal accomplishment (PA, 6 items) was reverse-scored and defined as low with a cutoff score ≥ 33, indicating a diminished sense of professional achievement. For descriptive purposes, we report the prevalence of high EE, high DP, and low PA separately in the Results section. The MBI has demonstrated good reliability and validity in Chinese healthcare samples [16, 17]; in the present study, Cronbach’s α values were 0.82 for EE, 0.79 for DP, 0.80 for reduced PA, and 0.85 for the overall scale.

Psychological traits were evaluated using the Eysenck Personality Questionnaire Short Scale, which measures neuroticism, psychoticism, and extraversion. Neuroticism was coded as a binary variable (yes/no) according to established cutoffs, while extraversion was categorized into introversion, ambiversion, and extraversion. Social support was measured using the Perceived Social Support Scale (PSSS), which classifies support into unobstructed (high/moderate social support) or impaired (low social support) [18]. Self-efficacy was assessed using the General Self-Efficacy Scale (GSES), which categorizes self-efficacy into four levels: markedly low, slightly low, moderately high, and extremely high [19]. For regression analysis, GSES was recoded as a binary variable: low self-efficacy (markedly low/slightly low) and high self-efficacy (moderately high/extremely high).

### Statistical analysis and data visualization

Data were analyzed using SPSS 26.0. Continuous data are presented as mean ± standard deviation (Mean ± SD), with group comparisons performed via one-way analysis of variance (ANOVA) with LSD post-hoc tests. Categorical data are presented as frequencies and percentages [n(%)], with group comparisons performed using the chi-square test. Univariate logistic regression was used to screen factors associated with burnout. Variables with *P* < 0.05 in univariate analysis were entered into multivariable binary logistic regression to identify independent associated factors. Collinearity was assessed using variance inflation factors (VIF); VIF < 5 was defined as no severe collinearity. Adjusted odds ratios (aOR) and 95% confidence intervals (CI) were calculated. A two-sided *P* < 0.05 was considered statistically significant. Data visualization was performed using GraphPad Prism 10. A forest plot was used to display unadjusted crude ORs and 95% CIs for subgroup analyses. All estimates in the forest plot were unadjusted.

## Results

### Study population and baseline characteristics

**Fig. 1:**
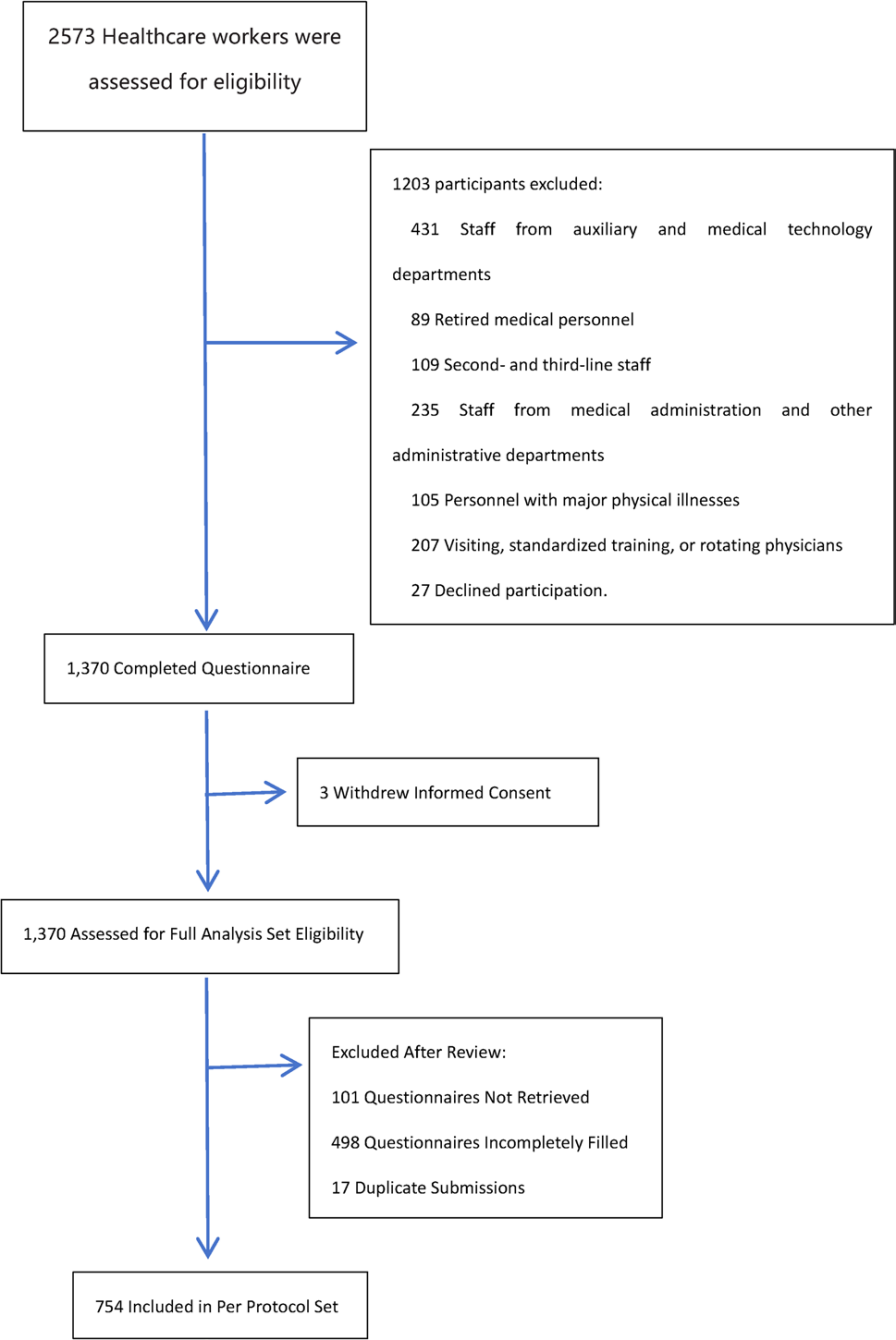
Flowchart of participant enrollment and selection. Flow diagram that systematically depicts the screening, enrollment, exclusion, and final analysis population of healthcare professionals in the study

A total of 2573 healthcare professionals underwent eligibility assessment; 1203 were excluded (auxiliary/technical staff, retired personnel, administrators, trainees, those with severe physical illnesses, non-participants), leaving 1370 who completed questionnaires. After excluding 3 who withdrew consent, 101 with uncollected data, 498 with incomplete responses, and 17 duplicate entries, 754 participants were included in the analytic sample (Fig. 1).

Baseline characteristics are summarized in Table 1. The overall burnout prevalence (total MBI score ≥ 50) was 7.3% (55/754), with 92.7% (699) reporting no burnout. By department, 29.7% (224) were from psychiatry and 70.3% (530) from non-psychiatry departments; by hospital, 39.9% (301) were from Affiliated Hospital of Jining Medical University, 30.4% (229) from Jining No.1 People’s Hospital, and 29.7% (224) from Jining Mental Health Center.

**Table 1:** Baseline Demographic and Psychological Characteristics of Participants (*n* = 754)

Project		Number of People	Percentage
Job Burnout	No	699	92.7%
	Yes	55	7.3%
Hospital	Jining Medical University Affiliated Hospital	301	39.9%
	Jining No.1 People’s Hospital	229	30.4%
	Jining Mental Health Hospital	224	29.7%
Gender	Male	249	33.0%
	Female	505	67.0%
Marital Status	Marital Status	619	82.1%
	Unmarried	130	17.2%
	Divorced	5	0.7%
Education Level	Technical Diploma	14	1.9%
	Associate Degree	97	12.9%
	Bachelor’s Degree	393	52.1%
	Master’s Degree	235	31.2%
	Doctoral Degree	15	2.0%
Professional Title	Junior	449	59.5%
	Intermediate	228	30.2%
	Associate Senior	55	7.3%
	Senior	22	2.9%
working years	≤ 5 Years	231	30.6%
	6–10 Years	320	42.4%
	11–20 Years	102	13.5%
	≥ 21 Years	101	13.4%
Age Group	20 ~ 29 Years	238	31.6%
	30 ~ 39 Years	395	52.4%
	40 ~ 49 Years	62	8.2%
	≥ 50 Years	59	7.8%
Interpersonal Relationships	Dissatisfied	3	0.4%
	Less Satisfied	12	1.6%
	Generally Satisfied	114	15.1%
	Relatively Satisfied	300	39.8%
	Satisfied	325	43.1%
Income Circumstances	Dissatisfied	110	14.6%
	Less Satisfied	74	9.8%
	Generally Satisfied	318	42.2%
	Relatively Satisfied	150	19.9%
	Satisfied	102	13.5%
Psychoticism	No	720	95.5%
	Yes	34	4.5%
Introvert-Extravert Dimension	Introversion	291	38.6%
	Ambiversion	192	25.5%
	Extraversion	271	35.9%
Neuroticism	No	495	65.6%
	Yes	259	34.4%
Social Support	Unobstructed	670	88.9%
	Impaired	84	11.1%
GSES	Markedly Low	5	0.7%
	Slightly Low	119	15.8%
	Moderately High	443	58.8%
	Extremely High	187	24.8%

The cohort was predominantly female (67.0%, 505) and married (82.1%, 619). Educational background included 52.1% (393) with a bachelor’s degree, 31.2% (235) with a master’s degree, 12.9% (97) with an associate degree, 2.0% (15) with a doctoral degree, and 1.9% (14) with a technical diploma. Professional titles were distributed as 59.5% (449) junior, 30.2% (228) intermediate, 7.3% (55) associate senior, and 2.9% (22) senior. Work experience included 42.4% (320) with 6–10 years, 30.6% (231) with ≤ 5 years, 13.5% (102) with 11–20 years, and 13.4% (101) with ≥ 21 years. Age categories were defined based on typical career stages in Chinese healthcare: early career (20–29 years), mid-career (30–39 years), late career (40–49 years), and senior career (≥ 50 years); the cohort was concentrated in mid-career (52.4%, 395) and early career (31.6%, 238).

Psychologically, 34.4% (259) of participants had neuroticism traits, 4.5% (34) had psychoticism traits, 38.6% (291) were introverted, 35.9% (271) were extroverted, and 25.5% (192) were ambiverted. Social support was unobstructed for 88.9% (670) and impaired for 11.1% (84). For self-efficacy, 58.8% (443) had moderately high scores, 24.8% (187) extremely high, 15.8% (119) slightly low, and 0.7% (5) markedly low. Regarding life satisfaction, 43.1% (325) were satisfied with interpersonal relationships and 42.2% (318) had neutral income satisfaction.

### Burnout prevalence and associated factors

**Fig. 2:**
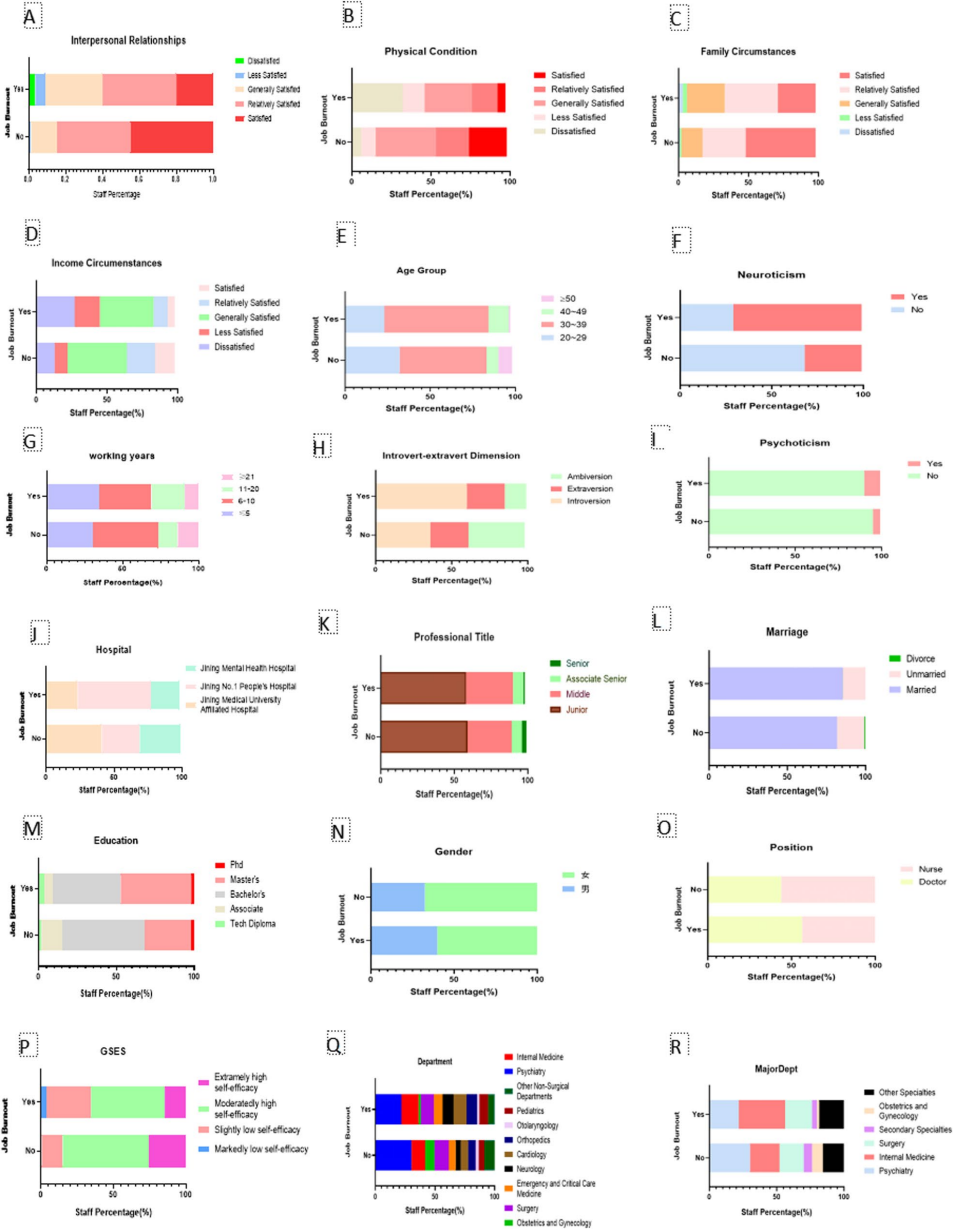
Figure 2 Stacked bar plots (Panels A–R) illustrating the distribution of covariates (satisfaction levels, personality traits, work-related factors, and sociodemographic characteristics) across burnout and non-burnout groups, complementing the univariate analysis in Table 2 and visualizing key correlates of burnout risk

Univariate analysis results are presented in Table 2; Fig. 2. Age, gender, marital status, professional title, education level, occupation, psychoticism, department, and work experience showed no significant associations with burnout. No significant difference in burnout prevalence was observed between doctors (54.5%) and nurses (43.6%)   . (OR = 0.606, 95% CI: 0.349-1.055, P > 0.05). 

**Table 2:** Univariate Analysis of Factors Associated with Occupational Burnout (*n* = 754)

	Job Burnout	Yes（%）	No（%）	chi-square value	OR（95%CI）	*P*-value
Age Group	20~29	13（23.6%）	225（32.2%）	6.388	1.006（0.726~1.395）	0.969
	30~39	34（61.8%）	361（51.6%）			
	40~49	7（12.7%）	55（7.9%）			
	≥ 50	1（1.8%）	58（8.3%）			
Hospital	Jining Medical University Affiliated Hospital	13（23.6%）	288（41.2%）	16.599	0.797（0.357~1.783）	0.581
	Jining No.1 People's Hospital	30（54.5%）	199（28.5%）		2.663（1.327~5.346）	0.006
	Jining Mental Health Hospital	12（21.8%）	212（30.3%）		reference	0.000
Gender	Male	22（40.0%）	227（32.5%）	1.305	1.427（0.975~2.090）	0.255
	Female	33（60.0%）	472（67.5%）			
Occupation	Doctor	31（56.4%）	307（43.9%）	3.192	0.606（0.349~1.055）	0.076
	Nurse	24（43.6%）	392（56.1%）			
Education	Tech Diploma	2（3.6%）	12（1.7%）	8.258	1.427（0.975~2.090）	0.067
	Associate	3（5.5%）	94（13.4%）			
	Bachelor’s	24(43.6%)	369(52.8%)			
	Master’s	25(45.5%)	210(30.0%)			
	Phd	1(1.8%)	14(2.0%)			
Marriage	Married	47(85.5%)	572(81.8%)	0.725	0.748(0.353 ~ 1.581)	0.446
	Unmarried	8(14.5%)	122(17.5%)			
	Divorce	0(0)	5(0.7%)			
Professional Title	Junior	32(58.2%)	417(59.7%)	0.895	0.984(0.682 ~ 1.419)	0.931
	Middle	18(32.7%)	210(30.0%)			
	Associate Senior	4(7.3%)	51(7.3%)			
	Senior	1(1.8%)	21(3.0%)			
Family Circumstances	Dissatisfied	2(3.6%)	8(1.1%)	0.925	0.599(0.458 ~ 0.783)	0.000
	Less Satisfied	2(3.6%)	9(1.3%)			
	Generally Satisfied	15(27.3%)	109(15.6%)			
	Relatively Satisfied	21(38.2%)	218(31.2%)			
	Satisfied	15(27.3%)	355(50.8%)			
Interpersonal Relationships	Dissatisfied	2(3.6%)	1(0.1%)	38.332	2.291(1.675 ~ 3.135)	0.000
	Less Satisfied	3(5.5%)	9(1.3%)			
	Generally Satisfied	17(30.9%)	97(13.9%)			
	Relatively Satisfied	22(40.0%)	278(39.8%)			
	Satisfied	11(20.0%)	314(44.9%)			
Income Circumstances	Dissatisfied	15(27.3%)	95(13.6%)	16.265	0.640(0.507 ~ 0.809)	0.000
	Less Satisfied	10(18.2%)	64(9.2%)			
	Generally Satisfied	21(38.2%)	297(42.5%)			
	Relatively Satisfied	6(10.9%)	144(20.6%)			
	Satisfied	3(5.5%)	99(14.2%)			
Physical Condition	Dissatisfied	18(32.7%)	45(6.4%)	52.775	0.488(0.382 ~ 0.622)	0.000
	Less Satisfied	8(14.5%)	66(9.4%)			
	Generally Satisfied	17(30.9%)	267(38.2%)			
	Relatively Satisfied	9(16.4%)	152(21.7%)			
	Satisfied	3(5.5%)	169(24.2%)			
Psychoticism	No	29(85.3%)	670(93.1%)	2.892	2.310(0.857 ~ 6.228)	0.098
	Yes	5(14.7%)	50(6.9%)			
Introvert-extravert Dimension	Introversion	33(60.0%)	258(36.9%)	14.6	0.509(0.355 ~ 0.729)	0.001
	Ambiversion	14(25.5%)	178(25.5%)			
	Extraversion	8(14.5%)	263(37.6%)			
Neuroticism	No	16(29.1%)	479(68.5%)	35.163	5.307(2.903 ~ 9.703)	0.000
	Yes	39(70.9%)	220(31.5%)			
Social Support	Impaired	12(21.8%)	72(10.3%)	6.833	0.411(0.207 ~ 0.816)	0.011
	Unobstructed	43(78.2%)	627(89.7%)			
GSES	Markedly low self-efficacy	2(3.6%)	3(0.4%)	19.648	2.170(1.430 ~ 3.293)	0.000
	Slightly low self-efficacy	17(30.9%)	102(14.6%)			
	Moderately high self-efficacy	28(50.9%)	415(59.4%)			
	Extremely high self-efficacy	8(14.5%)	179(25.6%)			
MajorDept	Psychiatry	12(21.8%)	212(30.3%)	8.178	reference	0.193
	Internal Medicine	19(34.5%)	153(21.9%)		2.194(1.034 ~ 4.654)	0.041
	Surgery	11(20%)	126(18%)		1.542(0.661 ~ 3.599)	0.316
	Secondary Specialties	2(3.6%)	43(6.2%)		0.822(0.177 ~ 3.804)	0.802
	Obstetrics and Gynecology	1(1.8%)	55(7.9%)		0.321(0.041 ~ 2.524)	0.280
	Other Specialties	10(18.2%)	110(15.7%)		1.606(0.673 ~ 3.834)	0.286
working years	≤ 5	19(34.5%)	212(30.3%)	5.002	0.953(0.719 ~ 1.265)	0.741
	6–10	19(34.5%)	301(43.1%)			
	11–20	12(21.8%)	90(12.9%)			
	≥ 21	5(9.1%)	96(13.7%)			

* Factors with Statistically Significant Association (*P* < 0.05). *Reference groups*: Hospital = Jining Mental Health Center, Occupation = Nurse, Neuroticism = No, Social support = Unobstructed, Interpersonal relationship = Satisfied

Statistically significant correlates of burnout included hospital affiliation: Jining No.1 People’s Hospital had a significantly higher burnout risk than Jining Mental Health Center (OR = 2.663, 95% CI:1.327–5.346, *P* < 0.05). Life satisfaction factors also showed strong associations: dissatisfaction with family circumstances (OR = 0.599, 95% CI:0.458–0.783, *P* < 0.001), interpersonal relationships (OR = 2.291, 95% CI:1.675–3.135, *P* < 0.001), income (OR = 0.640, 95% CI:0.507–0.809, *P* < 0.001), and physical condition (OR = 0.488, 95% CI:0.382–0.622, *P* < 0.001) were all linked to higher burnout. Psychological traits including introversion (OR = 0.509, 95% CI:0.355–0.729, *P* = 0.001) and neuroticism (OR = 5.307, 95% CI:2.903–9.703, *P* < 0.001) were significant risk factors. Social support served as a protective factor (OR = 0.411, 95% CI:0.207–0.816, *P* = 0.011), and low self-efficacy (OR = 2.170, 95% CI:1.430–3.293, *P* < 0.001) was associated with higher burnout rates.

### Prevalence of burnout and distribution of mbi dimensions

The mean scores of the three burnout dimensions were as follows: emotional exhaustion 14.80 ± 7.424, depersonalization 7.67 ± 5.478, and reduced personal accomplishment 11.80 ± 7.451. Burnout was defined as a total MBI score ≥ 50. High EE was 63.3% (477/754), high DP was 25.1% (189/754), and low PA was 2.3% (17/754). The overall prevalence of burnout was 7.3% (55/754).

Sensitivity analyses were performed using three distinct burnout definitions: total MBI ≥ 50, high emotional exhaustion (EE ≥ 27, 63.3%), and combined tri-dimensional criteria (EE ≥ 27 + DP ≥ 10 + PA ≤ 33, 5.8%). The patterns of departmental differences and associated factors remained consistent across all definitions, confirming the robustness of the main findings.

### Burnout dimensions across departments and occupations

**Fig. 3:**
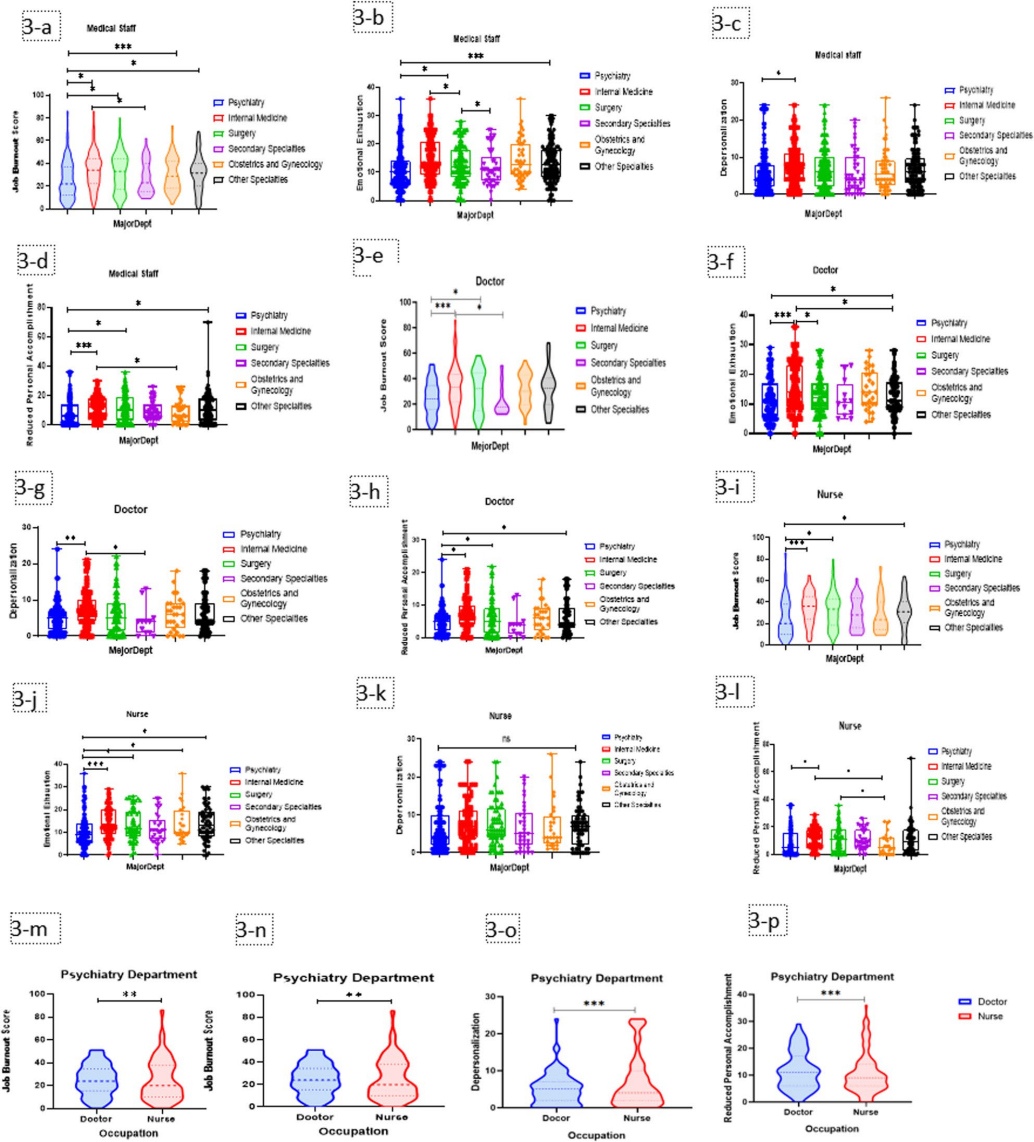
Figure 3 One-way ANOVA for total and three dimensions of occupational burnout. Violin/box plots (Panels a–p) comparing total burnout and its subdimensions across departments and professional roles (all staff, physicians, nurses, and psychiatry-specific subgroups), with statistical significance marked (*p < 0.05, **p < 0.01, ***p < 0.001)

ANOVA results for burnout dimensions are presented in Fig. 3. Among all healthcare professionals, total burnout scores were highest in internal medicine (33.99 ± 15.499) and lowest in psychiatry (24.98 ± 16.158), with a gradual decreasing trend across surgery, other clinical departments, obstetrics and gynecology, and subspecialties. All comparisons with internal medicine were statistically significant (*P* < 0.05).

Emotional exhaustion was highest in internal medicine (14.80 ± 7.424) and lowest in psychiatry (10.98 ± 7.261), with internal medicine scoring significantly higher than general surgery (*P* = 0.022) and subspecialties (*P* = 0.009). Depersonalization was highest in internal medicine (7.67 ± 5.478) and significantly lower in psychiatry (6.22 ± 6.178; *P* = 0.012 < 0.05). Reduced personal accomplishment was highest in internal medicine (11.80 ± 7.451) and lowest in psychiatry (8.74 ± 9.793), with psychiatry scoring significantly lower than internal medicine (*P* = 0.001), general surgery (*P* = 0.006) and other clinical departments (*P* = 0.012) (Fig. 3-d).

Among physicians, total burnout scores were highest in internal medicine (33.67 ± 16.583) and lowest in psychiatry (24.88 ± 13.508), with psychiatry scoring significantly lower than internal medicine (*P* < 0.001), general surgery (*P* = 0.024), obstetrics and gynecology (*P* = 0.048) and other clinical departments (*P* = 0.013). Emotional exhaustion was highest in internal medicine (15.48 ± 8.014) and obstetrics and gynecology (14.86 ± 6.704), with psychiatry (11.71 ± 7.037) scoring significantly lower (*P* < 0.05) (Fig. 3-h).

For nurses, total burnout scores were highest in internal medicine (34.31 ± 14.652) and general surgery (32.49 ± 16.845) and lowest in psychiatry (25.04 ± 17.722), with psychiatry scoring significantly lower than internal medicine (*P* < 0.001), general surgery (*P* < 0.001) and other clinical departments (*P* = 0.017). Depersonalization showed no significant interdepartmental differences (*P* > 0.05) (Fig. 3-l).

In the psychiatry department, significant differences were observed between physicians and nurses: nurses had slightly higher total burnout scores (25.04 ± 17.722 vs. 24.88 ± 13.308, *P* < 0.05) (Fig. 3-m and -p), significantly higher depersonalization (6.91 ± 7.030 vs. 5.09 ± 4.250, *P* < 0.001) and reduced personal accomplishment (8.95 ± 10.449 vs. 8.39 ± 8.662, *P* < 0.05); physicians had significantly higher emotional exhaustion (11.71 ± 7.037 vs. 10.54 ± 7.384, *P* = 0.043) (Fig. 3-n).

### Subgroup analyses and forest plot

**Fig. 4:**
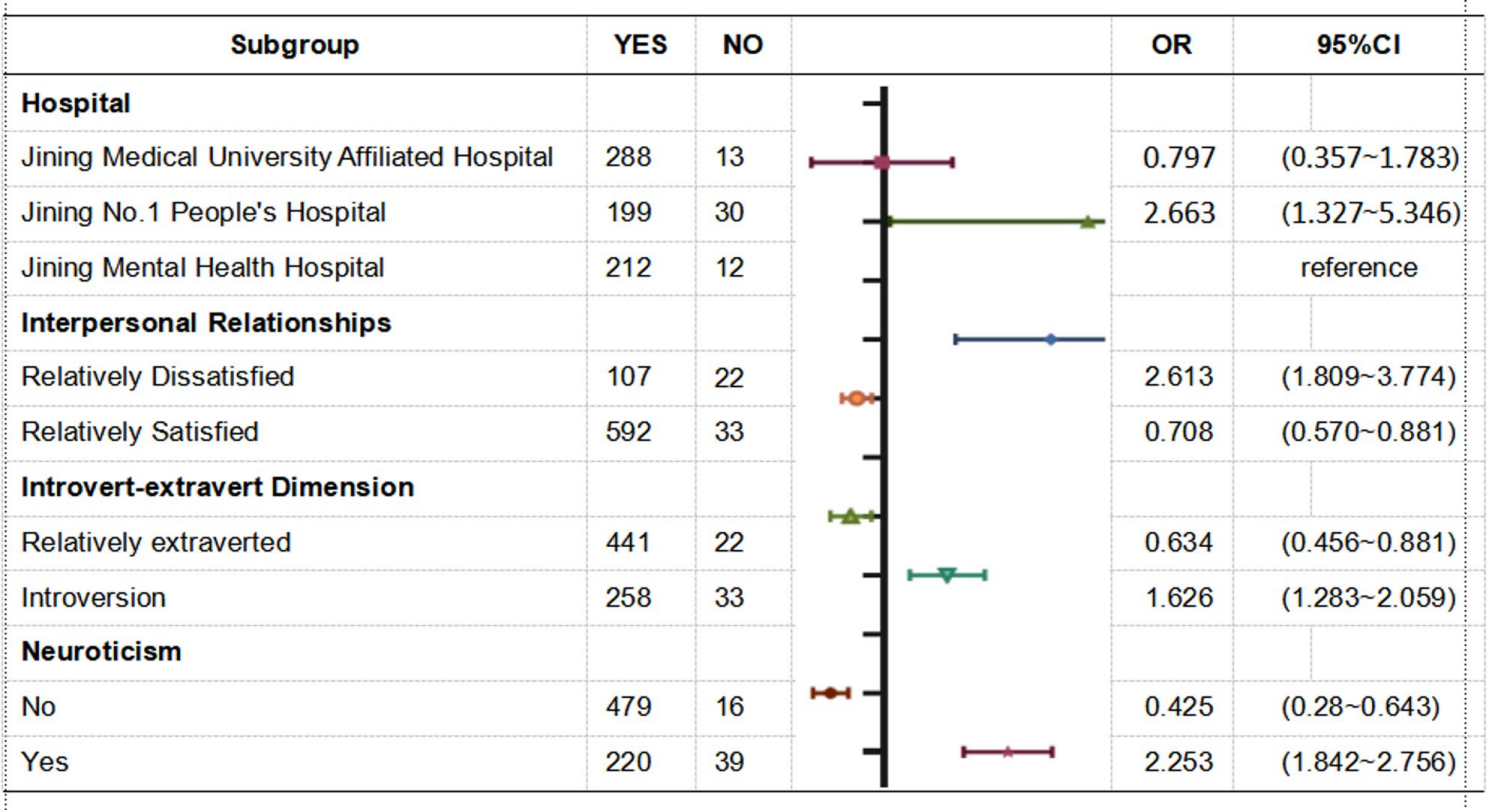
Subgroup Analyses of Occupational Burnout (Forest Plot). The vertical dashed line represents the null effect (OR = 1.0). Horizontal lines represent 95% confidence intervals. Factors with CIs not crossing 1.0 are statistically significant. All estimates were unadjusted crude ORs. Reference group: Jining Mental Health Hospital

Nine statistically significant factors from univariate analysis—hospital affiliation, interpersonal relationships, income, physical condition, family circumstances, neuroticism, introversion, social support, and GSES—were entered into a multivariable binary logistic regression model. Collinearity was assessed using variance inflation factors (VIF). All VIF values were between 1.09 and 2.32 (all < 5), indicating no severe collinearity among predictors. After adjustment, only four variables remained independently associated with occupational burnout (*P* < 0.05): hospital affiliation (Jining Medical University Affiliated Hospital vs. reference: aOR = 0.638, 95% CI: 0.272–1.498, *P* > 0.05; Jining No.1 People’s Hospital vs. reference: aOR = 2.423, 95%CI: 1.129–5.198,*P* = 0.023 < 0.05),interpersonal relationship(aOR = 0.422, 95%CI:0.184–0.972), introversion‑extraversion(aOR = 1.802, 95% CI:0.979–3.317), and neuroticism(aOR = 3.303, 95% CI:1.720–6.346). Subgroup analyses (Fig. 4, forest plot) were subsequently performed for these four independent risk factors. Factors with 95% CIs not crossing 1.0 were considered statistically significant. All estimates shown were unadjusted crude ORs and 95% CIs.

## Discussion

To our knowledge, this study is among the first multicenter investigations in China to compare burnout between internal medicine and psychiatry after adjusting for hospital affiliation and occupation. Our study provides novel adjusted evidence that burnout across all dimensions is significantly higher in internal medicine and lower in psychiatry after controlling for hospital and professional characteristics, challenging conventional perceptions of specialty-specific burnout risk. Independent associations identified for interpersonal dissatisfaction, neuroticism, and introversion further clarify actionable intervention targets, offering new insights to strengthen both local practice and the global evidence base.

This study systematically investigated the prevalence, independent associated factors, and interdepartmental/occupational differences of occupational burnout among 754 healthcare professionals in three tertiary hospitals in Jining, Shandong Province, accounting for key confounders (hospital affiliation, occupation) and using a clearly defined operational burnout criterion. The study addresses critical gaps in the existing literature by providing adjusted comparisons of burnout across clinical specialties (including psychiatry and internal medicine) and identifying independent factors associated with burnout via multivariable regression—an analytical approach rarely applied in previous Chinese studies of healthcare burnout. The key novel finding is that internal medicine is associated with significantly higher burnout across all dimensions than psychiatry in this population, even after controlling for hospital affiliation and professional composition, challenging the conventional assumption that psychiatric healthcare professionals face the highest burnout risk [10]. Additionally, this study is the first to report independent correlates of burnout in Jining’s healthcare workforce, highlighting interpersonal relationship satisfaction, introversion and neuroticism as key factors.

Direct comparison with previous studies was limited by heterogeneous MBI cutoffs, scoring algorithms, and study populations across publications. Regarding burnout prevalence and non-response bias, the overall burnout prevalence in this study was 7.3% (defined as a total MBI score ≥ 50). High EE was observed in 63.3% (477/754), high DP in 25.1% (189/754), and low PA in 2.3% (17/754). These rates are lower than the 15%–30% reported in previous Chinese multicenter studies [8, 16], but consistent with the 5%–10% in high-income countries [17]. This discrepancy may stem from three factors: regional differences in healthcare resources and management (Jining, eastern China, has more robust resources than central/western regions), exclusion of high-risk groups (auxiliary staff and trainees prone to burnout), and high unobstructed social support (88.9%), a known protective factor against burnout [18]. Owing to the anonymous survey design, individual data for non‑respondents were unavailable; no baseline comparison between respondents and non‑respondents was performed. Only department‑level distribution was recorded during questionnaire administration.This mild selection bias may slightly underestimate burnout prevalence (given internal medicine’s high burnout rates) but is unlikely to alter key findings.

A key finding was the significant departmental difference in burnout after multivariable adjustment: internal medicine staff exhibited the highest scores across all burnout dimensions (total burnout: 33.99 ± 15.499), whereas psychiatry staff had the lowest (total burnout: 24.98 ± 16.158), which runs counter to the conventional view that psychiatric professionals are at higher burnout risk [10]. This difference remained statistically significant after controlling for hospital affiliation and occupation. This result is consistent with previous studies reporting lower burnout in psychiatry and higher stress exposure in internal medicine, including heavy workload, complex chronic disease management, frequent contact with acute patients, and high administrative burdens. The lower burnout level in psychiatry may be explained by regular psychological resilience training, better stress-coping and emotional regulation skills, greater focus on doctor-patient communication, and lower patient volume and acuity compared with internal medicine. Meanwhile, Jining No.1 People’s Hospital showed a significantly higher burnout prevalence than Jining Mental Health Hospital( OR = 2.663, 95% CI:1.327–5.346, *P* < 0.05) in univariate analysis. This is likely due to its higher patient volume, more complex tertiary cases, and lower nurse-to-patient ratio [20, 21] compared with other hospitals. Distinct occupational variations were also observed within psychiatry independent of confounders: psychiatrists reported higher emotional exhaustion related to complex diagnosis and emotional labor, while psychiatric nurses showed higher depersonalization and lower personal accomplishment associated with routine care and acute outburst management [22, 23]. These findings support the need for role-specific supportive interventions even in departments with relatively low overall burnout.

Multivariable regression analysis revealed that multidimensional factors were associated with burnout, which could be categorized into two domains: psychological traits and interpersonal relationship satisfaction. These findings are consistent with recent global systematic reviews and meta-analyses reporting high prevalence of burnout among healthcare professionals and consistent modifiable correlates including psychological factors [24–26]. Neuroticism and introversion were significant psychological risk factors for burnout, in line with previous personality research indicating that neurotic individuals are more vulnerable to stress reactivity and introverts tend to exhibit weaker social support-seeking behaviors. Regarding life satisfaction, interpersonal relationship dissatisfaction showed the strongest association with burnout (OR = 2.291, 95% CI: 1.675–3.135, *P* < 0.001) [27]; given the interpersonal nature of healthcare work, conflicts with patients, colleagues, or family members may increase psychological distress, whereas positive relationships serve as a protective buffer. Notably, demographic characteristics including age, gender, and professional title were not significantly associated with burnout, which differs from some previous reports [28, 29]. This discrepancy may be attributed to the homogeneity of the study sample, which was dominated by young and middle-aged participants aged 30–39 years (52.4%) and those with junior professional titles (59.5%).

Targeted interventions addressing the observed departmental differences (highest burnout in internal medicine, lowest in psychiatry) are proposed across hospital, individual, and societal levels as hypothetical and testable strategies rather than evidence-based policies. At the hospital level, resource allocation may prioritize high-burnout departments such as internal medicine using evidence-supported approaches, including appropriate staffing adjustments, interdisciplinary chronic disease care teams to share clinical responsibilities, and digital tools such as AI-assisted electronic medical record systems to reduce administrative workload [30, 31], consistent with the view that organizational structure and resource allocation are closely related to burnout [20]. For internal medicine, the formation of sub-specialty teams may help reduce clinical complexity, and scheduling reforms may alleviate emotional exhaustion as reported in similar settings [13]. Successful practices from psychiatry, including regular resilience training and biweekly peer support sessions integrated into routine work, may be adopted across all departments [9]. A cross-departmental burnout monitoring system using the MBI may be conducted quarterly to identify high-risk groups with elevated emotional exhaustion [14], while compensation structures adjusted for workload and clearer career development pathways may help improve professional fulfillment among healthcare staff [28].

At the individual level, healthcare professionals in high-burnout departments may benefit from tailored psychological literacy training (stress management, emotional regulation, cognitive restructuring) through a combination of online modules and in-person workshops, drawing on evidence-based resilience skills from psychiatry [15]. Peer mentorship programs between psychiatric and internal medicine staff may facilitate the transfer of stress-coping strategies [9], and the development of social support networks through interdepartmental forums and family engagement initiatives may help buffer occupational stress [18]. For internal medicine providers, structured work–life boundary setting (e.g., limiting after-hours work communications) and regular self-care practices may help reduce emotional exhaustion [19]. At the societal level, potential supportive measures could include increased healthcare investment from authorities to address resource shortages in internal medicine [30], stronger legal protections for healthcare workers with clear dispute resolution mechanisms to alleviate stress related to doctor–patient conflicts [27], and expanded public mental health education to highlight the role of psychiatry in burnout prevention and reduce stigma [10].

Our study has several limitations. As a cross-sectional investigation, we cannot draw causal inferences or establish the temporal order of observed associations. The sample was restricted to three tertiary hospitals in Jining, which may restrict the generalizability of findings to other regions or healthcare settings. The use of self-reported data may introduce social desirability bias, and the low response rate (29.3%) may lead to selection bias that could affect prevalence estimates and associated factors. Comparability with previous studies is also limited by variations in measurement tools and cutoff criteria across the literature. Multiple pairwise comparisons were conducted, which may increase the risk of false-positive results; thus, findings should be interpreted with appropriate caution. Additionally, data were collected in 2018 (pre‑COVID‑19 pandemic), and the healthcare environment in China has changed substantially since then, further limiting the generalizability of our findings to the current clinical context.

Future research may adopt a multicenter longitudinal cohort design to clarify causal relationships. Additionally, integrating objective measures such as biological stress markers, electronic work hour records, and adverse event reports may enhance the comprehensiveness of assessment. Finally, future studies may validate the effectiveness of the proposed interventions through randomized controlled trials to support evidence-based and scalable policy development.

## Conclusions

Contrary to conventional assumptions, internal medicine was associated with significantly higher burnout levels and psychiatry with lower levels among healthcare professionals in Jining, after adjusting for hospital affiliation and occupational confounders. Hospital affiliation, interpersonal relationship, introversion-extraversion, and neuroticism were independent factors associated with burnout. Targeted public health interventions—including workload optimization in internal medicine and scaling resilience-building practices from psychiatry—may help mitigate burnout and strengthen healthcare workforce resilience. These findings provide novel, adjusted evidence for Chinese healthcare organizations and contribute to the global evidence base on healthcare worker burnout by identifying departmental differences and independent correlates in an understudied eastern Chinese population.

## Data Availability

The datasets generated and/or analyzed during the current study are available from the corresponding author on reasonable request. The raw data have been de-identified and stored in a password-protected electronic database with access restricted solely to the core research team. No personally identifiable information was collected to link responses to individual participants. Supplementary tables and figures are available with the revised manuscript and include participant comparison data, individual burnout dimension prevalence, and unadjusted burnout scores.

## Abbreviations

MBI: Maslach Burnout Inventory
ICD-11: International Classification of Diseases, 11th Revision
ICU: Intensive Care Unit
EE: Emotional exhaustion
DP: Depersonalization
PA: Reduced personal accomplishment
ANOVA: One-way analysis of variance
VIF: Variance inflation factors
aOR: Adjusted odds ratios
CI: Confidence intervals
PSSS: Perceived Social Support Scale
GSES: General Self-Efficacy Scale
